# On the Convergence of Stresses in Fretting Fatigue

**DOI:** 10.3390/ma9080639

**Published:** 2016-07-29

**Authors:** Kyvia Pereira, Stephane Bordas, Satyendra Tomar, Roman Trobec, Matjaz Depolli, Gregor Kosec, Magd Abdel Wahab

**Affiliations:** 1 Department of Electrical Energy, Systems and Automation, Ghent University, Zwijnaarde B-9052, Belgium; KyviadeFatima.ResendePereira@UGent.be; 2 Faculté des Sciences, de la Technologie et de la Communication, Université du Luxembourg, Luxembourg-Kirchberg L-1359, Luxembourg; Stephane.Bordas@uni.lu (S.B.); tomar.sk@iitkalumni.org (S.T.); 3 Department of Communication Systems, Jozef Stefan Institute, Ljubljana 1000, Slovenia; roman.trobec@ijs.si (R.T.); matjaz.depolli@ijs.si (M.D.); gkosec@ijs.si (G.K.); 4 Division of Computational Mechanics, Ton Duc Thang University, Ho Chi Minh City, Vietnam; 5 Faculty of Civil Engineering, Ton Duc Thang University, Ho Chi Minh City, Vietnam; 6 Soete Laboratory, Faculty of Engineering and Architecture, Ghent University, Technologiepark Zwijnaarde 903, Zwijnaarde B-9052, Belgium

**Keywords:** finite element analysis, fretting fatigue, convergence, stress analysis

## Abstract

Fretting is a phenomenon that occurs at the contacts of surfaces that are subjected to oscillatory relative movement of small amplitudes. Depending on service conditions, fretting may significantly reduce the service life of a component due to fretting fatigue. In this regard, the analysis of stresses at contact is of great importance for predicting the lifetime of components. However, due to the complexity of the fretting phenomenon, analytical solutions are available for very selective situations and finite element (FE) analysis has become an attractive tool to evaluate stresses and to study fretting problems. Recent laboratory studies in fretting fatigue suggested the presence of stress singularities in the stick-slip zone. In this paper, we constructed finite element models, with different element sizes, in order to verify the existence of stress singularity under fretting conditions. Based on our results, we did not find any singularity for the considered loading conditions and coefficients of friction. Since no singularity was found, the present paper also provides some comments regarding the convergence rate. Our analyses showed that the convergence rate in stress components depends on coefficient of friction, implying that this rate also depends on the loading condition. It was also observed that errors can be relatively high for cases with a high coefficient of friction, suggesting the importance of mesh refinement in these situations. Although the accuracy of the FE analysis is very important for satisfactory predictions, most of the studies in the literature rarely provide information regarding the level of error in simulations. Thus, some recommendations of mesh sizes for those who wish to perform FE analysis of fretting problems are provided for different levels of accuracy.

## 1. Introduction

Fretting happens when two contacting surfaces are normally loaded and subjected to small amplitude oscillatory relative movement. This amplitude generally varies from 5 to 100 µm [1], but it can be as low as, or even below, 1 µm [2]. Due to its cyclic characteristic and the high stresses gradient in the vicinity of contact, fretting may lead to unexpected failure due to fretting fatigue, being responsible for the premature failure of many common mechanical assemblies, such as bolted joints, shrink-fitted shafts, and dovetail joints. As a consequence, it has been an important research topic that has been vastly studied in the literature [3,4,5,6]. 

**Figure 1:**
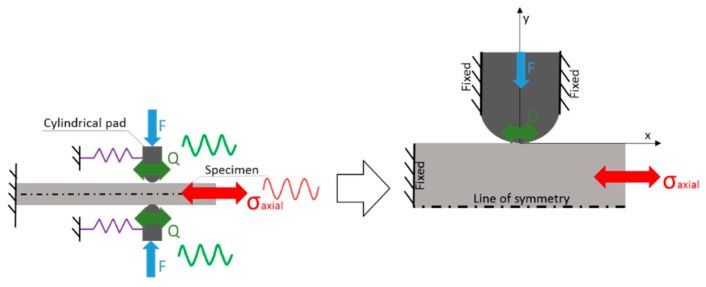
Cylinder-on-plane: Scheme of a fretting fatigue experimental set-up.

In order to evaluate the effects of different variables (surface finishing, coefficient of friction, normal load, relative slip amplitude, among others) on the characteristics of fretting, different laboratory tests are generally used. One of the most common is a cylinder-on-pad configuration, as illustrated in Figure 1. In this set-up, two cylindrical pads are maintained in contact with a flat specimen through the application of a constant clamping or normal force, *F*. The specimen is fixed at one end and the other end is subjected to an oscillatory bulk stress *σ_axial_*. On application of the bulk stress, the compliance springs transmit an oscillatory tangential force, *Q*, at the pads. Generally, the tangential load *|Q|* is smaller than the product of the normal load, *F*, by the coefficient of friction µ and the contact is divided into two regions: A stick zone and a slip region. In the early 1970s, Nishioka and Hirakawa [7] had already used this configuration to study the effects of slip amplitude in the fatigue strength of specimens. Even in recent research, this test set-up is still very common. For instance, Pierres et al. [8] proposed a combined numerical and experimental approach to simulate fretting fatigue crack growth of 2D and 3D configurations. A similar methodology was used by Luke et al. [9], however, they were interested in simulating crack initiation using different damage parameters and they used laboratory tests to validated their predictions.

Under fretting conditions, the fatigue limit of a material may be shortened by up to 50% [10]. It is known that, in this case, the crack growth phase is significantly different from plain fatigue propagation phase, due to the influence of contact stresses distributions on the crack and vice versa [11]. This contact-crack interaction is particularly important for cracks’ length smaller than the magnitude of the contact zone dimension [12] and must be taken into account. For a cylinder-on-plane configuration, the stress and strain field in the specimen can be analytically estimated by a combination of the normal pressure distribution *p*(*x*) (due to the normal force, *F*) and surface traction *q*(*x*) (due to the tangential and bulk loads, *Q* and *σ_axial_*, respectively). However, these solutions are valid under a series of conditions, such as infinite and idealized bodies, elastic material properties, and loading conditions, among others. In addition, the stress field near the contact region is variable, multiaxial and non-proportional [13], which provides extra complexity to the phenomena.

Fretting fatigue is a complex phenomenon due the stick-slip zone at the contact interface. This complex phenomenon is not well understood and a recent research report [14] has questioned the applicability of the analytical solution (Cattaneo–Mindlin problem) to the stick-slip problems. In the analytical solution, the superimposing of shear stress due to normal load and due to fatigue load is a linear approximation and ignores the effect of interaction between both loads. Furthermore, recent laboratory measurements [15,16] indicated that the transition from ‘static’ to ‘dynamic’ friction (stick-slip) can be described by classical fracture mechanics singular solutions of shear cracks, rather than by Coulomb law. This motivates us to investigate whether or not stress singularity takes place at the stick-slip zone in fretting conditions.

Numerical methodologies have become an interesting option to evaluate stresses at contact and its impact on fretting fatigue lifetime. In this regard, the finite element analysis (FEA) has been widely used over the past few decades. For instance, McVeigh and Farris [17] used finite element analysis to study the influence of the bulk loading *σ_axial_* on the contact stresses distributions, and compared the results with analytical approximations, validating the latter. Tur et al. [18] treated the problem considering the effects of plasticity on the contact stress distribution for a Titanium material and analyzed the impact of plastic deformations on the size of the stick zone and peak stresses. They concluded that the plastic zone started at the trailing edge (the edge of the largest slip zone) and that the effects of contact stresses decayed rapidly as the distance from the contact increased.

The focus of this paper is to recognize the existence of stress singularity at the stick-slip zone in fretting fatigue conditions using FEA. In order to do that, a finite element model of a fretting test configuration (cylindrical pad and flat specimen) was created and stresses at the contact interface were monitored and compared with analytical solutions for different mesh sizes and fretting contact conditions. 

The paper is organized in the following way. Firstly, the analytical solutions of the contact stresses used as references in this study are described in Section 2. Then the finite element models are constructed and details of them are provided in Section 3. Finally, the results are presented and discussed in Section 4 and conclusions are drawn in Section 5.

## 2. Analytical Solutions

In this section, we first present the Hertzian solutions for the pressure distribution at the contact interface of a cylinder and a flat surface under normal load. Then, we consider the effect of combined normal and tangential loads, and, finally, we shall present solutions for the effect of bulk stresses on fretting fatigue conditions.

### 2.1. Hertzian Solutions for the Pressure Distribution

As discussed by Johnson [19], the contact pressure distribution, *p*(*x*), due to the normal clamping force, *F*, between the elastic pad and elastic specimen, can be calculated analytically if the following contact conditions hold:

1. Contact surface profiles are smooth, continuous and nonconforming;
2. Small strains at contact region;
3. Bodies can be approximated as a semi-infinite elastic half-space near the contact zone;
4. Frictionless contact.

In this case, the contact pressure, *p*(*x*), is elliptical at a distance, *x*, from the center of the contact zone (see Figure 1) and is given by [19]:

(1) $$p\left(x\right)=p_{max}\sqrt{1-{\left(\frac{x}{a}\right)}^{2}}\;\text{and}\;p_{max}=\sqrt{\frac{FE^{*}}{t\pi R}}$$

where *p_max_* is the maximum contact pressure at the center of the contact; *R* is the combined curvature; and *E*^∗^ is the combined modulus of elasticity. Both *R* and *E*^∗^ can be defined as:

(2) $$\frac{1}{R}=\frac{1}{R_{1}}+\frac{1}{R_{2}}$$

(3) $$\frac{1}{E^{*}}=\frac{1-\nu_{1}^{2}}{E_{1}}+\frac{1-\nu_{2}^{2}}{E_{2}}$$

where *E_i_*, for *i* = 1,2 are the Young’s Modulus and *ν_i_*, for *i* = 1,2 are the Poisson’s ratio for the first and second bodies, respectively. The flat specimen can be considered as a cylinder with an infinitely large radius *R*_1_ = ∞ and the combined curvature, *R*, becomes equal to the radius of the surface of the pad *R*_2_.

Considering that contact should occur only inside the loaded area, and, also, the fact that all contact regions must be in compression, the semi-contact width, *a*, and the applied load, *F*, are related by:

(4) $$a=2\sqrt{\frac{FR}{t\pi E^{*}}}$$

where *t* is the thickness of cylinder pad. The elastic deformation of the surfaces results in a rectangular contact region of area equal to *2a* × *t*.

### 2.2. Solutions for Combined Normal and Tangential Loads

**Figure 2:**
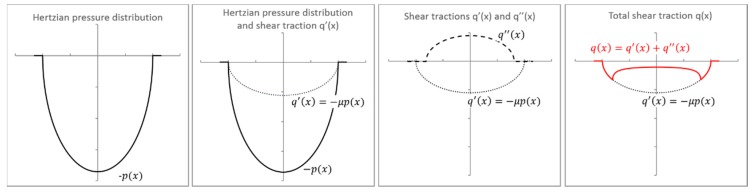
Illustration of the components of shear traction distributions.

When studying fretting, it is necessary to consider, not only the normal loading condition, but also the effect of the tangential frictional force, *Q*. The Coulomb friction law can be used to model the contact shear traction, *q*(*x*), at an arbitrary position, *x*, as a function of the normal contact pressure, *p*(*x*), and the coefficient of friction, *µ*. If *Q* is smaller than the product of *µ* and the normal load, *F*, the contact region will be divided into two different zones: Stick and slip, in which the width of the stick zone is denoted by *c*. In this case, the contact shear traction can be seen as combination of a pressure distribution and two superposed shear tractions, *q’*(*x*) due to *p*(*x*) and *q’’*(*x*) due to *Q*, as shown in Figure 2.

The complete expression for the shear traction *q*(*x*) can be written as [12,19]:

(5) $$q\left(x\right)=\{\begin{array}{cc} -\mu p_{max}\sqrt{1-{\left(\frac{x}{a}\right)}^{2}}, & c\leq \left|x\right|\leq a\\ -\mu p_{max}\left[\sqrt{1-{\left(\frac{x}{a}\right)}^{2}}-\frac{c}{a}\sqrt{1-{\left(\frac{x}{c}\right)}^{2}}\right], & \left|x\right|<c \end{array}$$

where $\frac{c}{a}=\sqrt{1-\frac{Q}{\mu F}}$.

### 2.3. Effect of Bulk Load σ_axial_ on Contact Shear Traction

According to Hills and Nowell [12], the contact shear traction presented above can be adjusted for the presence of bulk stresses *σ_axial_*. This causes an eccentricity to the solution presented in Section 2.2, and for the case of negative tangential load, it can be written as [12]:

(6) $$\left(x\right)=\{\begin{array}{cc} -\mu p_{max}\sqrt{1-{\left(\frac{x}{a}\right)}^{2}}, & c\leq \left|x\right|\leq a\\ -\mu p_{max}\left[\sqrt{1-{\left(\frac{x}{a}\right)}^{2}}-\frac{c}{a}\sqrt{1-{\left(\frac{x+e}{c}\right)}^{2}}\right], & \left|x+e\right|<c \end{array}$$

where $\frac{c}{a}=\sqrt{1-\frac{Q}{\mu F}}$ and $e=\frac{a\sigma_{axial}}{4\mu p_{max}}$.

**Figure 3:**
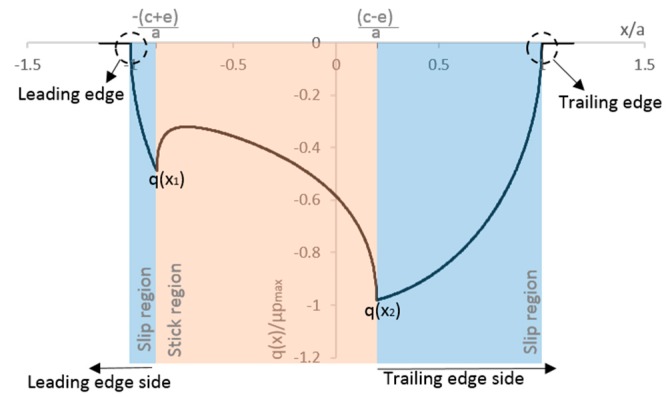
Typical normalized shear traction distribution at contact interface (*Q* = 155.165 N, *σ_axial_* = 100 MPa, *p_max_* = 185.03 MPa, *µ* = 0.4 and *a* = 0.467 mm).

Figure 3 shows a typical normalized shear traction distribution for fretting fatigue specimen using Equation (6). Note that, based on this distribution, it is possible to determine the size of the stick and slip zones and also the peak values of shear stresses. For this paper, we monitored two peak values of shear tractions q(x_1_) and q(x_2_), at the leading edge and at trailing edge sides (the edge of the largest slip zone [20]), respectively. 

### 2.4. Effect of Bulk Load σ_axial_ on Subsurface Stresses

**Figure 4:**
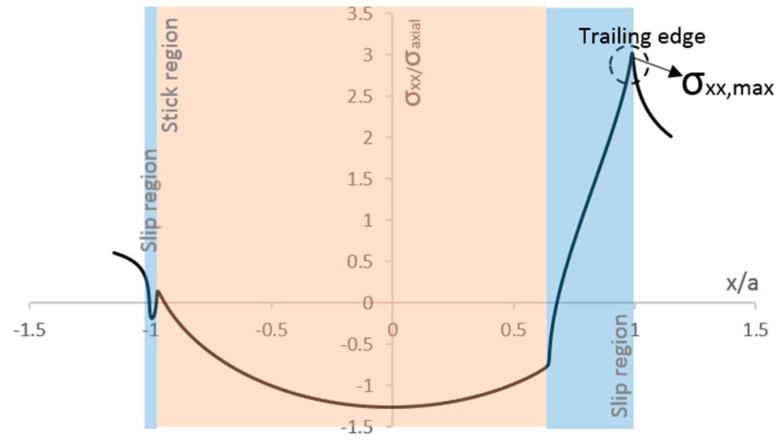
Typical normalized principal stress σ_xx_ distribution at contact interface, obtained from finite element analysis (*Q* = 155.165 N, *σ_axial_* = 100 MPa, *p_max_* = 185.03 MPa, *µ* = 0.85 and *a* = 0.467 mm).

In his literature review, Mutoh [4] mentioned studies showing that fretting fatigue cracks, which propagate to material final ruptures, originate in the edge of the contact area (*x* = *a*), while small arrested cracks are initiated near the maximum shear traction *q(x_2_)*. Other research [12,21,22] has also pointed out that the principal crack initiates near the trailing edge (*x* = *a*). The reason for that may be related to the contribution of the principal stress *σ_xx_* in the stress state at the contact interface. As discussed by Szolwinski and Farris [23], studies showed that the sharp peak in tangential stresses *σ_xx,max_*, at trailing edge of the contact region (see Figure 4), might play a significant role on fretting fatigue crack initiation.

There are analytical solutions for subsurface elastic stresses, *σ_xx_*, as function of *x* for a given normal and tangential loads (*F* and *Q*) and coefficient of friction, *µ*, in the slip zone [12,19,24]. For instance, Szolwinski and Farris [24] provided an analytical solution for the stress distribution, *σ_xx_*, treating the problem as a superposition of individual stress components, caused by the normal pressure distribution and surface tractions, *q’*(*x*) and *q’’*(*x*).

Although the addition of the bulk stress *σ_axial_* brings some extra complexity to the problem, there are still some simplified equations to estimate stresses at contact. McVeigh and Farris [17] adjusted the analytical solution from Szolwinski and Farris [24] by adding bulk stress in the distribution of *σ_xx_*. Szolwinski and Farris [23], based on the work done by McVeigh and Farris [17], provided a simplified equation to estimate the maximum peak stress *σ_xx,max_* as:

(7) $$\sigma_{xx,max}=2p_{max}\sqrt{\frac{\mu Q}{F}}+\sigma_{axial}$$

## 3. Finite Element Model: Cylinder Pad on Flat Specimen

A parametric 2D finite element model was created in ABAQUS^®^ and an analysis of the fretting cycle was performed, aiming to study the model response to different mesh sizes. Three values of coefficients of friction were considered (0.3, 0.85 and 2.0). These variable values of coefficient of friction (COF) allowed us to study different configurations of stick-slip regions and, therefore, to simulate different fretting scenarios.

**Figure 5:**
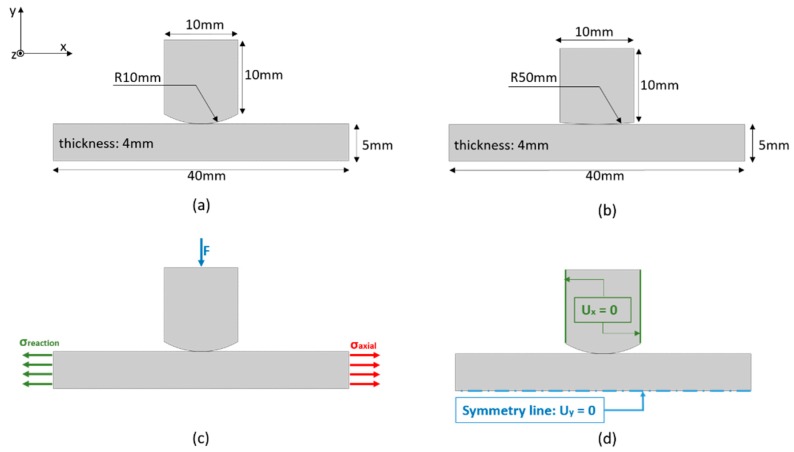
Details of the models: (**a**) dimensions of FE fretting fatigue model with 10 mm pad radius; (**b**) dimensions of FE fretting fatigue model with 50 mm pad radius; (**c**) loading and (**d**) boundary conditions.

The model details, such as geometry, material properties, mesh details, boundary conditions and loading history, are presented here. Two FE models were developed and their dimensions and boundary conditions are shown in Figure 5. The models were composed of only two parts: A pad and a specimen, which represents half of the experimental set-up, due to its symmetry. In order to check the influence of different geometries, the radius of the pad was also variable in those models, and two values were chosen: 50 mm and 10 mm. Both parts were made of aluminum 2420-T3, having material properties which are summarized in Table 1. We did not consider any plasticity effect in this study, only an elastic material response. Stress analysis was carried out by applying a normal load (*F* = 543 N) and oscillatory axial and reaction stresses to the specimen, reflecting a fretting cycle.

The master-slave algorithm in ABAQUS^®^ was used to describe the contact behavior and the Lagrange multiplier formulation was used to define the tangential behavior of the contact pair. The surface-to-surface and finite sliding options were used to define the contact interaction.

**Figure 6:**
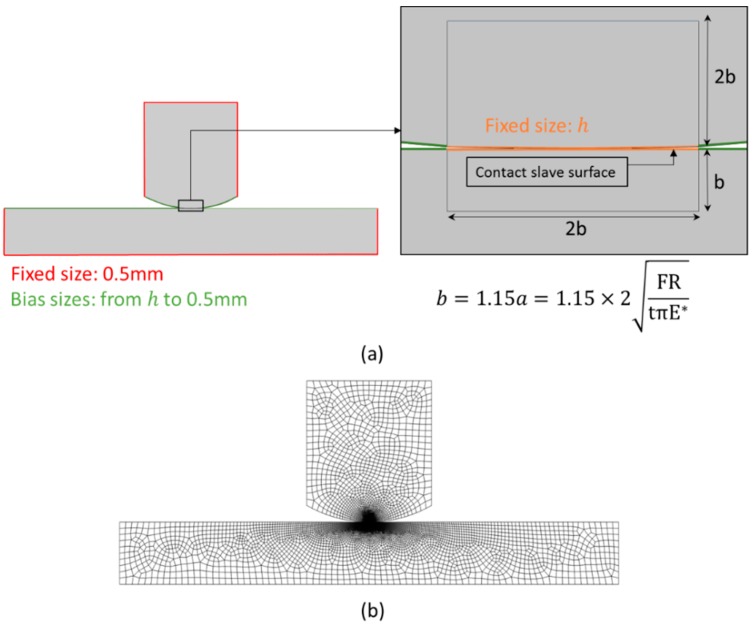
Details of the used mesh: (**a**) partition of model and the edges seeding (element size *h* varied from values: 20 µm, 10 µm, 5 µm, 2.5 µm, 1.25 µm, 0.625 µm, 0.3125 µm) and (**b**) an illustration of the model with pad radius of 10 mm and mesh size of 2.5 µm.

A 2D quadrilateral, 4-node (bilinear), plane strain, reduced integration element (CPE4R) was used in both models. Different mesh sizes were considered at the contact interface and increased as the distance from the contact region increased. In order to create a fine mesh at the contact region, the models were partitioned and the edges were seeded. The values of the mesh element size along the contact region varied according to the following list: 20, 10, 5, 2.5, 1.25, 0.625 and 0.3125 µm. Details of the seeding used to generate the mesh and also of the model partition dimensions are shown in Figure 6. The partition dimensions were dependent on the radius of the pad, being calculated based on the semi-contact width, *a*, from Equation (4). An illustration of one of the meshes used in this study is also presented in Figure 6.

Due to the symmetry of the problem, the bottom of the specimen (representing the axial centerline of the specimen) was restricted from vertical movement in the *y* direction (*U_y_* = 0). The sides of the pads were restricted from horizontal movement in the *x* direction (*U_x_* = 0) and the Multiple point constraints (MPC) tie constraint was also used at the top surface of the pad to guarantee that it would not rotate due to the applied concentrated load, *F*.

The effect of the compliance spring and tangential load *Q* were modeled as a cyclic reaction stress (*σ_reaction_*). This reaction stress is obtained as:

(8) $$\sigma_{reaction}=\sigma_{axial}-2\frac{Q}{bt}$$

where *b* is the specimen width (*b* = 10 mm); *t* is the specimen thickness (*t* = 4 mm). The values of *Q* and *σ_axial_* are obtained from experimental data (see Table 2). For this study, they are taken from the experimental set-up FF1 in Reference [25].

**Figure 7:**
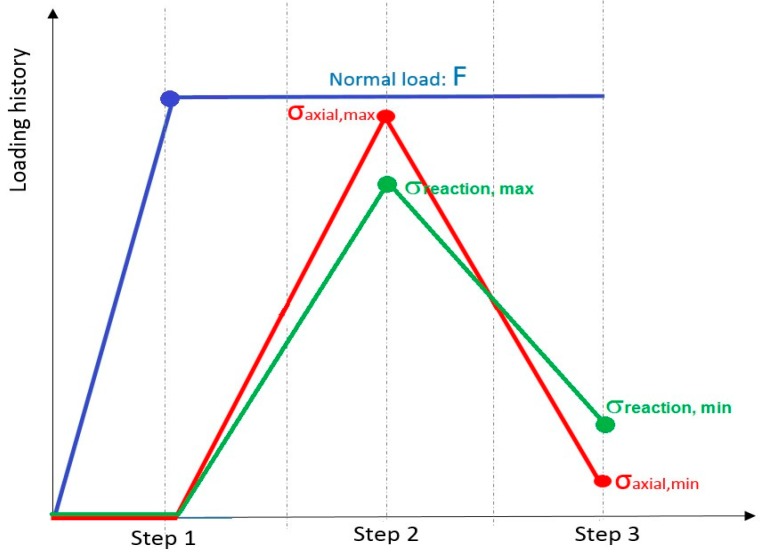
Fretting simulation: Loading variation as a function of time.

In order to simulate fretting fatigue conditions, the loads are applied in three steps (see Figure 7), with adaptive time steps in ABAQUS^®^. In the first loading step, the top pad was pressed against the specimen surface by a normal load *F* = 543 N and this compressed condition was held constant until the end of the cycle. Then, both axial and reaction maximum stresses were applied to the sides of the specimen (values are presented in Table 2). Finally, in the third loading step, both axial and reaction minimum stresses were applied.

## 4. Results and Discussion

In order to recognize a singularity’s presence, the methodology presented by Sinclair [27] was adopted. Accordingly, the element size in the models was successively systematically halved for a sequence of seven analyses and the magnitude of maximum stress values was examined. The following stress components were monitored at the maximum axial loading condition (end of loading step 2): The contact shear traction peak at trailing edge side *q*(*x*_1_) and at leading edge side *q*(*x*_2_) (see Figure 3) and the peak tangential stress in the *x* direction, *σ_xx,max_* (see Figure 4). The influence of the mesh size on the values of the ratios between stick and slip zones sizes (*c*/*a*) is also considered here. The slip zone size, *c*, is obtained by measuring the position in the contact that have non-zero values of slip and the contact width, *a*, is obtained by the position in the x direction of the edges of the contact region, both calculated from ABAQUS^®^.

The results of various stress components and for the ratios between stick and slip zones sizes (*c*/*a*) are presented in Table 3, for different values of coefficient of friction and different radius of cylindrical pad. FEA results were also compared with analytical solutions (Equations (6) and (7), presented in Section 2.3 and Section 2.4, respectively). The values of shear traction at trailing and leading edges seem to converge to the analytical solution for all values of coefficient of friction. Regarding the values of peak tangential stress *σ_xx,max_*, they seem to converge, but to a different value than the estimate from Equation (7). This is reasonable, since this equation provides only an approximate value of *σ_xx,max_*. Note that the non-dimensional parameter (*c*/*a*) also converges on the analytical solution for all values of coefficient of friction and pad radius. 

To examine convergence in Table 3, the relative error between FE and analytical solutions was considered. If those errors do not decrease with successively refined analysis, divergence occurs and the presence of a singularity is detected. 

### 4.1. Stress Singularity Check: Influence of Mesh Size on Stress Components

In order to analyze the influence of mesh size on the contact shear traction, the analytical solution was chosen as a reference value. The relative error between FE and analytical solutions (*e_rel,an_*) was calculated as:

(9) $$e_{rel,an}=\left|\frac{\phi_{max}^{a}-\phi_{max}^{i}}{\phi_{max}^{a}}\right|$$

where $\phi_{max}^{i}$ is the maximum variable output (contact shear stress, maximum tangential stress or ratio between stick slip zone sizes) in the *ith* model and $\phi_{max}^{a}$ in the analytical solution (see Table 3). 

**Figure 8:**
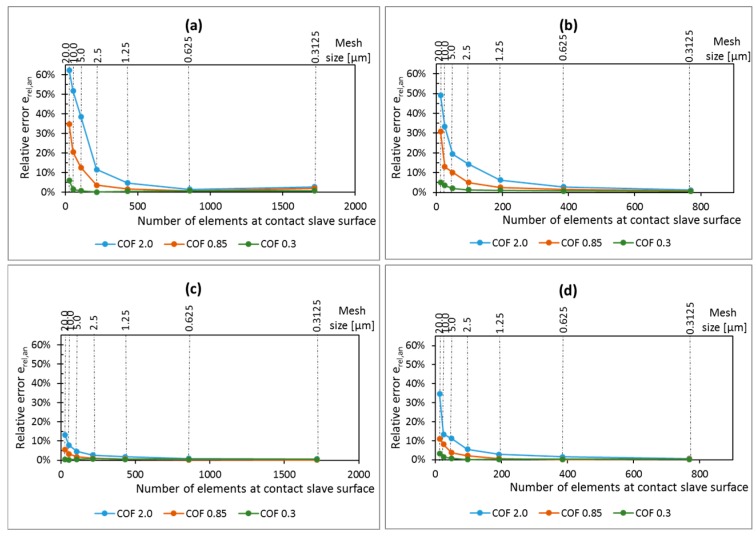
Mesh convergence curves for the peak values of shear stress near the trailing edge for different pad geometries: (**a**) Pad radius of 50 mm and (**b**) pad radius of 10 mm and also for the peak values of shear stress near the leading edge, considering different pad geometries; (**c**) pad radius of 50 mm and (**d**) pad radius of 10 mm.

Higher coefficient of friction implies stronger gradients in the stress distribution, and singularities are expected to happen for higher values of coefficient of friction. The relative error between FE and analytical solutions for the contact shear traction stress component for different coefficients of friction and pad radius are presented in Figure 8. The results show that the error is decreasing as the mesh size reduces, independent of the value of coefficient of friction and pad radius. Thus, the analysis is converging, even if only slowly, and no singularity was found for any of the tested loading conditions, pad radius, and coefficients of friction.

Moreover, it can also be seen that the rate of convergence is dependent on the coefficient of friction for both cases of pad geometry. As different values of coefficient of frictions represent different loading conditions (various sizes of stick zone in comparison with the contact dimension), one might conclude that the rate of convergence of the solution depends on the loading condition. For the smallest coefficient of friction, a relative coarse mesh (around 20 µm) at the contact is sufficient for obtaining reasonable accurate shear stresses at contact, with relative error smaller than 10% for all analyzed cases. However, for higher coefficients of friction, the rate of convergence reduces and it is necessary to use relative fine meshes to guarantee reasonable results. For instance, for coefficient of friction equal to 2.0, a mesh size of 1.25 µm is enough to guarantee that the relative error on the shear traction peak is smaller than 10% for all analyzed cases. However, for the same coefficient of friction and a mesh size of 5 µm, the error can increase to almost 40%, for the contact shear traction peak at leading edge. 

**Figure 9:**
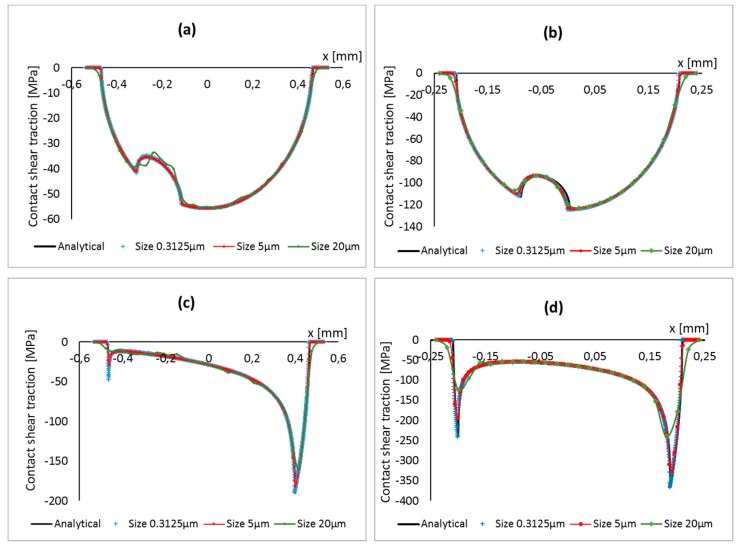
Contact shear traction at contact interface for different mesh sizes, pad radius and coefficients of friction: (**a**) Pad radius of 50 mm and COF 0.3; (**b**) pad radius of 10 mm and COF 0.3 (**c**) pad radius of 50 mm and COF 2.0; and (**d**) pad radius of 10mm and COF 2.0.

The dependence of the rate of convergence on the coefficient of friction can be further investigated by analyzing the contact shear traction at contact interface. As can be seen in Figure 9, for the case of high coefficient of friction, the contact shear traction distribution has very sharp peaks at both leading and trailing edges, justifying the necessity of a very fine mesh to accurately capture those steep gradients. It is clear that for a higher coefficient of friction, a very fine mesh size is required in order to achieve convergence. This is due to the fact that the value of the friction coefficient affects the contact stress distribution and the larger the coefficient of friction, the steeper the stress gradient. The value of coefficient of friction also affects the stick-slip zone size, which is an important parameter to determine a suitable mesh size as explained in Section 4.2.

As discussed before, the peak stress *σ_xx,max_*, seems to converge to a different value than the estimate from Equation (7). Therefore, in order to study the convergence of the results of the, instead of considering the analytical solution as a reference, the maximum stresses between two subsequent mesh refinements were used to calculate the relative error *e_rel_* as:

(10) $$e_{rel}=\left|\frac{\phi_{max}^{i+1}-\phi_{max}^{i}}{\phi_{max}^{i+1}}\right|$$

where $\phi_{max}^{i}$ is the maximum variable output (contact shear stress, maximum tangential stress or ratio between stick slip zone sizes) in the *i*th model and $\phi_{max}^{i+1}$ in the *(i* + 1*)*th model. 

**Figure 10:**
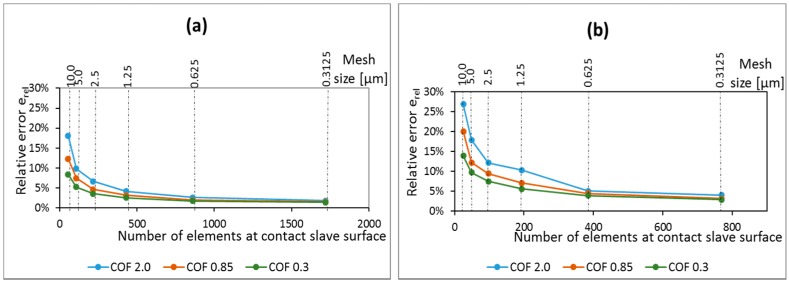
Mesh convergence curves for the maximum tangential stress for different cases of coefficient of friction and different pad radius: (**a**) 50 mm and (**b**) 10 mm.

The results of the relative error between two consecutive mesh sizes for the maximum tangential stress are presented in Figure 10. The results also show that the error reduces with successively refined analysis, independent of the value of coefficient of friction or pad radius. Thus, once again, one may conclude that the analysis is converging and no singularity’s presence was found.

**Figure 11:**
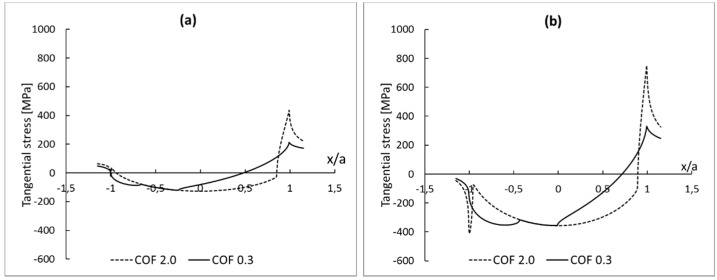
Tangential stress at contact interface as function of normalized contact width. Results from FEA model with mesh size equal to 0.3125 µm for different pad radius: (**a**) 50 mm and (**b**) 10 mm.

Additionally, some comments regarding the convergence rate can be made. For a mesh size of 0.625 µm, the relative error is around 5% for all analyzed scenarios, and results can be considered to be good [28]. As for the shear traction component, the convergence rate of the maximum tangential stress depends upon the coefficient of friction. Again, for the smallest coefficient of friction, the convergence rate is the highest. This dependency may be further investigated by checking the tangential stress distribution at contact, as shown in Figure 11.

As mentioned in Reference [29], increasing the pad radius causes a reduction on the peak contact pressure and increases the contact width. Therefore, for the same loading conditions, the contact pressure distribution has a steeper gradient for pads with smaller radius. As discussed by the authors of [12,19], and presented in Section 2, the analytical distribution of shear stress at contact can be seen as a superposition of contact pressure distribution and two shear tractions (Figure 2). Thus, it is expected that the gradient of the distribution of the tangential stresses at the trailing edge is higher for the model with smaller pad radius. The smaller contact width for smaller pad radius also implies higher peak values of tangential stresses in a smaller area (Figure 11). Therefore, for the same loading conditions, a finer mesh is necessary to properly to capture this changes in the model with a smaller pad radius.

It can also be observed in Figure 11 that the peak values and, therefore, the gradients of the distribution of the tangential stresses are smaller for low coefficients of friction. Consequently, the convergence rate, for the model with a 10 mm pad radius, is slower than for the model with a 50 mm pad radius, as can be seen in Figure 10. This impact of geometry on convergence rate is expected, as the smallest radius implies smaller contact region (Equation (4)) for the same loading condition. It also implies higher peaks of tangential stresses in a smaller area. Thus, for the same level of accuracy, a finer mesh is required in the model with a smaller pad radius. 

### 4.2. Fretting Fatigue Convergence Map

As pointed out by Ainsworth and Oden [30], although aware of the existence of numerical errors, the analyst is seldom interested in quantifying them. In fretting fatigue, the quality of a simulation is generally assessed by visual comparison between finite element results and analytical solutions [18,31,32], and information regarding the error is rarely provided [22]. The accuracy of the fretting contact stress calculations is of significant importance, as these stresses impact directly on the crack propagation phase. Therefore, estimating errors for those stresses is of great interest to ensure an accurate analysis.

**Figure 12:**
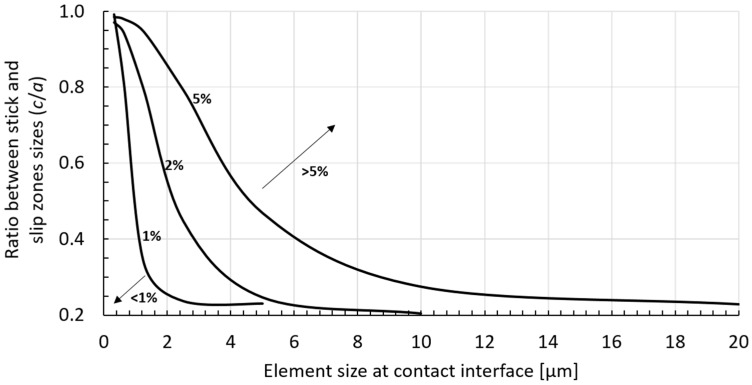
Fretting fatigue convergence map: Stick–slip ratio (c/a) as function of the element size in the contact zone for different numerical accuracies (1%, 2% and 5%).

Aiming to help researchers to easily determine the required element size for their finite element analysis for a given stick-slip ratio and desired accuracy, a ‘fretting fatigue convergence map’ was produced and is presented in Figure 12. This map was constructed by plotting the stick–slip ratio (c/a) against the element size in the contact zone for different numerical accuracies (1%, 2% and 5%) and may be used as a reference for choosing the element size in FEA of fretting fatigue (cylinder on plane configuration).

## 5. Conclusions

In this paper, we investigate the singularity’s presence in fretting fatigue stresses distributions at a contact interface. Different scenarios were studied: Three different coefficients of friction (replicating different loading conditions) and two different pad geometries (radius of 50 mm and 10 mm). For the considered loading conditions and coefficient of frictions, we could not find any indications that a singularity happens as the mesh becomes denser, our results all converged as the element size reduced. Additionally, the convergence rate of the finite element models was discussed as a function of the coefficient of friction. We have confirmed that the convergence rate is smaller for higher coefficients of friction. This means that, for a fixed element size, the level of error in the analysis depends on the loading condition. Therefore, it is recommended that the analyst perform a mesh convergence study for each of his loading condition of interest, as it may impact the accuracy of results. Considering all scenarios that we have studied, a choice of element size of 0.625 µm at contact provided the smallest relative error for all variables, being around, or even smaller than, 5% and producing satisfactory results. A ‘fretting fatigue convergence map’ was also constructed, providing information on the required element size for a specific stick–slip ratio and different levels of accuracy.

## Figures and Tables

**Table 1:** Material properties for aluminum 2420-T3 [10,11].

E	Modulus of Elasticity [GPa]	72.1
*ν*	Poisson’s ratio	0.33
*σ*_0.2_	Yield Strength [MPa]	506 ± 9

**Table 2:** Values of maximum and minimum σ_reaction_ and σ_axial_, based on data from experimental test FF1 from Reference [26].

Steps	*σ_axial_* [MPa]	*σ_reaction_* [MPa]	Q [N]
Step 2 (maximum values)	100	92.2	155.165
Step 3 (minimum values)	10	17.8	−155.165

**Table 3:** FEA results and analytical solution for different coefficients of friction, different pad radius and different mesh sizes at the contact surface.

	Mesh size [µm]	Contact Shear Traction at Leading Side q(x_1_) [MPa]		Contact Shear Traction at Trailing Side q(x_2_) [MPa]		Maximum Tangential Stress *σ_xx,max_* [MPa]		c/a	
		R = 50 mm	R = 10 mm	R = 50 mm	R = 10 mm	R = 50 mm	R = 10 mm	R = 50 mm	R = 10 mm
**COF: 0.3**	**20**	38.81	107.02	53.72	119.93	167.49	208.72	0.167	0.136
	**10**	40.72	108.69	54.11	122.34	182.87	242.57	0.200	0.136
	**5**	41.01	110.40	54.23	123.19	192.96	268.70	0.206	0.174
	**2.5**	41.30	111.21	54.38	124.29	200.09	290.50	0.211	0.207
	**1.25**	41.47	111.44	54.24	124.33	205.33	307.81	0.212	0.211
	**0.625**	41.54	111.97	54.27	124.39	208.99	320.33	0.212	0.212
	**0.3125**	41.57	111.99	54.27	124.44	212.00	329.94	0.211	0.213
	**Analytical**	**41.29**	**112.68**	**53.99**	**124.11**	**208.35**	**342.28**	**0.218**	**0.218**
**COF: 0.85**	**20**	24.89	113.00	112.64	209.21	222.91	279.65	0.702	0.727
	**10**	30.33	142.02	115.31	216.27	254.12	349.91	0.779	0.750
	**5**	33.31	146.77	117.18	226.39	274.64	398.69	0.788	0.779
	**2.5**	36.73	155.01	118.06	230.10	287.93	440.60	0.805	0.799
	**1.25**	37.54	158.98	118.50	233.62	297.38	474.40	0.806	0.804
	**0.625**	38.34	160.91	118.89	234.83	303.26	496.58	0.808	0.808
	**0.3125**	38.80	162.08	119.01	235.81	308.06	513.08	0.808	0.809
	**Analytical**	**38.09**	**163.18**	**119.20**	**235.12**	**283.90**	**507.82**	**0.811**	**0.815**
**COF: 2.0**	**20**	17.53	123.20	165.85	240.56	275.64	322.49	0.893	0.667
	**10**	22.50	162.01	175.64	318.97	336.52	441.20	0.905	0.864
	**5**	28.59	195.58	181.98	327.15	373.08	537.49	0.910	0.895
	**2.5**	41.18	207.99	185.62	347.98	399.72	612.33	0.920	0.911
	**1.25**	44.39	227.64	187.36	357.64	417.17	683.24	0.918	0.917
	**0.625**	45.85	236.01	189.13	362.74	428.46	719.75	0.921	0.920
	**0.3125**	47.72	239.73	189.43	366.06	436.21	750.50	0.921	0.921
	**Analytical**	**46.52**	**242.70**	**190.72**	**368.12**	**382.10**	**725.56**	**0.925**	**0.926**
